# Kostenaspekte und erstattungsrechtliche Voraussetzungen der Phagentherapie

**DOI:** 10.1007/s00103-025-04065-x

**Published:** 2025-05-13

**Authors:** Dennis Häckl, Jutta Dillschneider, Christian Elsner

**Affiliations:** 1 Institut für öffentliche Finanzen und Public Management, Universität Leipzig, Grimmaische Str. 12, 04109 Leipzig, Deutschland; 2 PwC Legal, Mannheim, Deutschland; 3 Zentrum für künstliche Intelligenz, Universität zu Lübeck, Lübeck, Deutschland

**Keywords:** Kostenerstattung, Wirtschaftlichkeit, Antibiotika, Krankenhausapotheke, GMP, Reimbursement, Economic efficiency, Antibiotics, Hospital pharmacy, GMP

## Abstract

Die Phagentherapie gilt als vielversprechender Ansatz zur gezielten Behandlung bakterieller Infektionen. In Deutschland ist sie bislang nicht als reguläre medizinische Therapieform zugelassen. Der Artikel untersucht die wirtschaftlichen und erstattungsrechtlichen Herausforderungen der Phagentherapie. 2 Herstellungsansätze werden unterschieden: zum einen die patientenunabhängige, industrielle Produktion standardisierter Phagenpräparate, die durch Skaleneffekte potenziell Kosten senken könnte, jedoch aufgrund von Fixkosten, GMP-Anforderungen und der wirtsspezifischen Wirkung Unsicherheiten birgt, zum anderen die individuelle Herstellung in (Krankenhaus‑)Apotheken, die mittels magistraler Zubereitung patientenspezifische Phagenmischungen ermöglicht, jedoch Produktions- und logistische Aufwände sowie Qualitätsmanagement erfordert. Einen wesentlichen Kostenfaktor stellt zudem die Diagnostik zur Identifikation bakterieller Erreger dar, die zusätzliche Investitionen erfordert.

Die Istsituation in Deutschland zeigt, dass Phagentherapeutika bislang im Rahmen individueller Heilversuche verordnet und vergütet werden. Sie sind weder als reguläre Arzneimittel noch als Erstlinientherapie etabliert. Ausnahmeregelungen wie die Potenzialmethode (§ 137c SGB V) und die Notstandsversorgung (§ 2 Abs. 1a SGB V) ermöglichen unter bestimmten Bedingungen eine Kostenerstattung, erfordern aber Begründungen und schaffen Transaktionskosten.

Der Artikel gibt Handlungsempfehlungen zur Förderung der Phagentherapie, darunter Parallelen zur Vergütung der CAR-T-Zell-Therapie mit Zusatzentgelten für Rezepturarzneimittel; für Fertigarzneimittel werden erfolgsabhängige Vergütungsmodelle sowie eine evidenzbasierte Bewertung diskutiert. Zur Schaffung wirtschaftlicher Anreize werden staatliche Förderinstrumente benötigt.

## Abstract (en)

Phage therapy is considered a promising approach for the targeted treatment of bacterial infections. It has not yet been approved as a regular form of medical therapy in Germany. This article examines the economic and reimbursement challenges of phage therapy as a targeted treatment option for bacterial infections. Two production approaches are distinguished. On the one hand, patient-independent, industrial production of standardized phage therapies could potentially lower costs through economies of scale, yet is associated with uncertainties due to fixed costs, requirements for Good Manufacturing Practice (GMP), and host-specific activity. On the other hand, individual production in (hospital) pharmacies enables patient-specific phage mixtures via magistral compounding but requires production, logistical efforts, and quality management. A significant cost factor is also the diagnostics needed for the identification of bacterial pathogens, which requires additional investments.

The current situation in Germany shows that phage therapeutics are so far prescribed and reimbursed only as part of individual treatment attempts; they are neither established as regular medicinal products nor as first-line therapy. Exceptions such as the “Potential Method” (§ 137c SGB V) and emergency supply (§ 2 Abs. 1a SGB V) allow for reimbursement under certain conditions, but require justifications and generate transaction costs.

This article provides recommendations for promoting phage therapy, including drawing parallels to the reimbursement of CAR-T cell therapy with additional fees for patient-specific therapeutics; for ready-to-use products, outcome-based reimbursement models and evidence-based assessments are discussed. To create economic incentives, state funding instruments are needed.

## Hintergrund

Trotz ihres Potenzials zur Behandlung von bakteriellen Infektionen steht die Phagentherapie vor Herausforderungen bei der Integration in Gesundheitssysteme, insbesondere aufgrund regulatorischer und erstattungsrechtlicher Rahmenbedingungen, die wiederum auch im Kontext des Herstellungsprozesses und Anwendungsbereichs zu bewerten sind [1]. In Bezug auf die Herstellung und Bereitstellung von Phagentherapeutika wird regulatorisch und durch die Wirkweise von Phagentherapeutika bedingt zwischen zwei Hauptansätzen differenziert: patientenunabhängig im Voraus hergestellte standardisierte Präparate sowie individuell auf Abruf hergestellte Phagenmischungen in Krankenhausapotheken. Beide Ansätze haben unterschiedliche wirtschaftliche und rechtliche Implikationen in Bezug auf Aufwand, Erstattungswege und Wirksamkeit.

Der Beitrag analysiert die wirtschaftlichen Aspekte der Phagentherapie durch eine strukturierte Unterscheidung zwischen industrieller und individueller Herstellung, um die verschiedenen Kostenfaktoren zu identifizieren. Ziel ist es, eine fundierte Grundlage für die Bewertung der Wirtschaftlichkeit und Erstattungsfähigkeit dieser Therapieform zu schaffen. Nach Überlegungen zur Kostenentstehung bei Phagentherapeutika werden bestehende Vergütungsmechanismen und regulatorische Rahmenbedingungen in Deutschland vorgestellt. Sodann werden mögliche Wege zur Implementierung einer regelhaften Erstattung von Phagentherapeutika diskutiert. Der Beitrag schließt mit einem Ausblick und einem Fazit.

## Überlegungen zur Kostenentstehung bei Phagentherapeutika

Die Implementierung der Phagentherapie erfordert eine differenzierte Kostenanalyse, die zwischen dem patientenunabhängigen (industriellen) und individuellen Herstellungsansatz unterscheidet. Zudem sind Kosten für Diagnostik zu berücksichtigen.

### Industrielle Herstellung der Phagentherapeutika

Die industrielle Produktion standardisierter Phagenpräparate („off-the-shelf“) basiert auf Phagenbibliotheken, die eine Vielzahl von bakteriellen Erregern abdecken sollen. Relevante Kostenfaktoren sind:

1. *Produktionskosten:* Herstellung großer Mengen von Phagenpräparaten aus zu etablierenden Phagensammlungen unter standardisierten Bedingungen, einschließlich der Einhaltung regulatorischer Anforderungen gemäß § 13 Abs. 1 AMG (Arzneimittelgesetz);
2. *Lagerkosten:* Sicherstellung einer stabilen Lagerung, um die biologische Aktivität der Phagen zu gewährleisten. Hier sind vor allen Dingen die genügende Vorhaltung und die Vernichtung bei Ablauf der Präparate wesentliche Kostentreiber, was auch ein effektives Qualitätsmanagement erfordert;
3. *Distributionskosten:* Transport der Phagenpräparate zu klinischen Einrichtungen oder Apotheken unter Einhaltung spezifischer Lagerungs- und Transportbedingungen, insbesondere Temperaturkontrollen.

Die industrielle Produktion von Phagenpräparaten ermöglicht Skaleneffekte, indem größere Mengen unter standardisierten Bedingungen hergestellt werden, wodurch die Produktionskosten pro Einheit sinken [2]. Diese Effekte treten insbesondere bei vergleichsweise hohen Fixkosten auf, etwa durch die Anforderungen an die Gute Herstellungspraxis (GMP) in Produktionsstätten oder den Einsatz automatisierter Prozesse. Allerdings bringt die flächendeckende Versorgung mit Phagenpräparaten wirtschaftliche Herausforderungen mit sich, da die Vorhaltung umfangreicher Phagenbanken und die Produktion potenziell nicht wirksamer Präparate aufgrund der hohen Wirtsspezifität und der „Moving-Target“-Problematik (durch ständige Weiterentwicklung der Bakterien) mit Unsicherheiten verbunden sind. Für kleinere und mittelgroße Krankenhäuser mit begrenzten Fallzahlen sowie für pharmazeutische Unternehmen, die Phagentherapeutika industriell herstellen möchten, könnten wirtschaftliche Anreize fehlen. Eine regelhafte Vergütung im Rahmen einer Erstlinientherapie könnte die Attraktivität und wirtschaftliche Tragfähigkeit dieser Therapieform erhöhen.

### Individuelle Herstellung der Phagentherapeutika

Ein weiterer Ansatz ist die magistrale Zubereitung in Krankenhausapotheken, wie sie beispielsweise in Belgien 2018 rechtlich verankert wurde. Diese erlaubt Apotheken, Phagenlösungen auf Verordnung von Ärzten individuell, aber nach Monografien, d. h. nach standardisierter Herstellungsanleitung, herzustellen, woran anschließend ein Nutzennachweis vorgenommen werden könnte. Monografien basieren auf Standardrezepturen, deren therapeutische Sinnhaftigkeit und Zusammensetzung vorab geprüft wurden.

Eine ähnliche Integration der Phagentherapie in Deutschland könnte durch die Aufnahme von Phagenmischungen in das Arzneibuch oder das Neue Rezeptur-Formularium (NRF) erfolgen. Sind solche Rezepturen vorhanden, muss die Apotheke sich gem. § 6 ApBetrO an diese halten. Dazu benötigt die Apotheke gem. § 2a ApBetrO ein Qualitätsmanagementsystem nach dem Stand der Technik. Sie benötigt hingegen nicht eine Herstellungserlaubnis nach § 13 Abs. 1 AMG. Die Herstellung in Krankenhausapotheken erfordert dazu die Prüfung und stetige Qualitätskontrolle gemäß ApBetrO. Hier ist insbesondere § 11 Abs. 1 ApBetrO zu berücksichtigen, der vorschreibt, dass die verwendeten Inhaltsstoffe auf ihre ordnungsgemäße Qualität zu prüfen sind. Dieser Ansatz stellt eine begrenzte Lösung dar. Sowohl in Bezug auf die Gewährleistung der Arzneimittelsicherheit als auch die Haftung des Krankenhausträgers für die Krankenhausapotheke bzw. des Inhabers der Offizin-Apotheke ist auch die Apothekenherstellung aufgrund des einzuhaltenden Qualitätsmanagementsystems mit Aufwänden, Investitionen sowie Haftungsrisiken verbunden. Die Kostenfaktoren umfassen:

1. *Isolierungs- und Auswahlkosten:* Kosten für die Isolierung des Erregers zur Identifikation spezifischer Phagen und für die Auswahl der geeigneten Phagen aus Phagenbibliotheken oder De-novo-Isolierung (Isolierung bisher unbekannter Phagen aus natürlichen Quellen). Hier sind teils im Krankenhaus nicht gängige Verfahren im Kontext einer Vorhaltung klassischer mikrobiologischer Labore erforderlich;
2. *Produktionskosten:* Herstellung begrenzter Mengen zur Behandlung einzelner Patienten bzw. Patientengruppen entsprechend dem individuellen Bedarf in speziellen Laborumgebungen, was für einzelne Krankenhäuser hohe Aufwände erfordert;
3. *logistische Herausforderungen:* Hier entsteht ein Zeitaufwand für die Herstellung und Lieferung innerhalb eines engen Zeitrahmens von wenigen Stunden bis zu einem Tag, um den klinischen Anforderungen gerecht zu werden. Je nach Verteilung und Schwerpunktbildung von Apothekenleistungen in einem Verbund kann dies auch mit erhöhten Transportkosten im Vergleich zu vorab gefertigten Phagenpräparaten verbunden sein.

Die Kosten der individuellen Phagenherstellung können im Kontext anderer personalisierter Therapien, wie z. B. der CAR-T-Zell-Therapie, betrachtet werden. Letztere verursachte aufgrund des individuellen Herstellungsprozesses pro Patient Kosten von bis zu 320.000 € in den vergangenen Jahren zulasten der Gesetzlichen Krankenversicherung (GKV; [2]). Studien wie die von Ran et al. zeigen jedoch, dass Skaleneffekte durch Optimierung der Produktion – beispielsweise durch Installation weiterer Maschinen in einem Reinraum bei höherer Produktionskapazität – eine signifikante Senkung der Kosten ermöglichen. Diese Überlegungen sind auch auf die Phagentherapie übertragbar, die durch die Nutzung natürlicher Ressourcen und im Gegensatz zu anderen Zelltherapieverfahren mit vergleichsweise einfachen Produktionsprozessen grundsätzlich kostengünstiger gestaltet werden könnte. Dennoch erfordern der Aufwand für die Isolation spezifischer Phagen, die patientenindividuelle Herstellung und die Logistik eine *Kosten-Nutzen-Analyse*, insbesondere im Vergleich zu standardisierten Phagenpräparaten. Die Literatur und eigene Recherchen zeigen, dass bislang detaillierte Schätzungen zu den Produktionskosten für Phagenpräparate fehlen und eine Forschungslücke darstellen [1]. Zu untersuchen ist, ob durch steigende Produktionszahlen bei CAR-T-Zell-Therapien oder Phagentherapie Skaleneffekte und dadurch geringere Kosten auftreten. Kostensenkend könnten innovative Ansätze zur Optimierung der Produktion sein. Durch die Automatisierung manueller Prozesse könnten Produktionskosten gesenkt und die Verfügbarkeit der Therapien erhöht werden. Einige wissenschaftliche Arbeiten, wie z. B. von Metanat et al. [3], zeigen auf, dass verschiedene Ansätze in der Zelltherapeutikaherstellung deutlich unterschiedliche Kostenaspekte bedeuten können. Diese Erkenntnis lässt sich bei größeren Produktionsansätzen sicherlich auch auf die Phagenproduktion übertragen.

### Diagnostik

Die erfolgreiche Anwendung von Bakteriophagen erfordert eine eindeutige Identifikation der bakteriellen Infektionserreger und deren Empfindlichkeit gegenüber spezifischen Phagen. Daher stellen Kosten für diagnostische Verfahren einen relevanten Faktor dar. Kosten ergeben sich aus mehreren Prozessschritten im klinischen und mikrobiologischen Laborumfeld.

Gegenüber einer traditionellen Antibiotikatherapie erfordert die Phagentherapie eine gezielte Diagnostik zur Bestimmung der Spezies bzw. der bakteriellen Stämme [4]. Hierbei können auch moderne Verfahren wie Next Generation Sequencing (NGS) eingesetzt werden, die jedoch Investitions- und Betriebskosten erfordern. Ein weiterer Kostenfaktor ist die Testung der Phagenempfindlichkeit des bakteriellen Erregers mittels standardisierter mikrobiologischer Verfahren. Diese ist arbeitsintensiv und weist bei geringen Patientenzahlen höhere Kosten pro Patient auf, verglichen mit Verfahren, bei denen die Testung über automatisierte Plattformen erfolgt. Eine derartige Automatisierung würde jedoch Investitionen in die technische Infrastruktur bedingen [5].

## Istanalyse zur aktuellen Kostenerstattung in Deutschland – Sonderfälle sowie Ausnahmetatbestände

Leistungen der Gesetzlichen Krankenversicherung unterliegen dem Wirtschaftlichkeitsgebot, d. h., gem. § 12 Abs. 1 SGB V müssen die Leistungen „ausreichend, zweckmäßig und wirtschaftlich sein; sie dürfen das Maß des Notwendigen nicht überschreiten“. Zu überlegen ist hierbei, wie die Wirtschaftlichkeit nachgewiesen werden kann. Ein Nachweis der Wirksamkeit und Unbedenklichkeit der Leistung wird als notwendiger, aber noch nicht hinreichender Hinweis für Wirtschaftlichkeit angesehen.

In Deutschland wurden Phagentherapeutika bislang allein im Rahmen von individuellen Heilversuchen verordnet und vergütet [1]. Hierbei werden patientenindividuell hergestellte Phagenpräparate unter der direkten Verantwortung eines Arztes eingesetzt, wobei die Kostenübernahme nicht standardisiert ist und individuelle Verhandlungen erfordert.

Zu prüfen sind Wege, die Vergütung von Phagentherapeutika als (noch) nicht zugelassene Arzneimittel zu ermöglichen. Dies ist angesichts des Aufwandes und der Zeitdauer bis zu einer Zulassung zumindest kurzfristig ein Weg, Phagentherapeutika nutzbar zu machen. Nachfolgend werden aktuelle Vergütungsmöglichkeiten aufgezeigt, wobei die Kostenerstattung für Diagnostik ausgeblendet wird.

### Potenzialmethode § 137c Abs. 3 SGB V

Die stationäre Vergütung in Deutschland erfolgt primär auf Grundlage des SGB V, des Krankenhausentgeltgesetzes (KHEntgG) und der Fallpauschalenvereinbarung (DRG-System, Diagnosis Related Groups). Neue Untersuchungs- und Behandlungsmethoden (NUB) müssen, um im stationären Bereich erstattungsfähig zu sein, nach § 137c SGB V im Fall einer schwerwiegenden Erkrankung und bei Fehlen einer wirksamen Standardtherapie ein Potenzial zur erfolgreichen Therapie mitbringen. Ist dies gegeben und fehlt ein Negativvotum des Gemeinsamen Bundesausschusses (G-BA), so besteht ein Kostenerstattungsanspruch als Rezepturarzneimittel auch ohne vollen Nutzennachweis [6]. Es geht hier darum, Patienten vielversprechende Heilungs- und Behandlungschancen zeitnah auch außerhalb von Studien zu gewähren, auch wenn deren Nutzen noch nicht auf hohem Evidenzlevel belegt ist [7]. Voraussetzung ist, dass es sich um eine neue Behandlungsmethode handelt, die das Potenzial einer erforderlichen Behandlungsalternative hat [8]. Was dies im Einzelnen bedeutet, ist in der Rechtsprechung und Literatur noch umstritten. Es wird vertreten, dass zumindest Empfehlungen in S1-Leitlinien oder einzelne (nichtrandomisierte) Studien vorliegen müssen, um ein solches Potenzial annehmen zu können (vgl. Zusammenstellung bei Stallberg, a. a. O., S. 337 [8]). Das Bundessozialgericht (BSG) hat zuletzt gefordert, dass eine bestehende Evidenzlücke zumindest durch eine Studie geschlossen werden könne [9]. Weitere Voraussetzung ist, dass eine Standardbehandlung mit hinreichenden Chancen zur Linderung und/oder Heilung nicht vorhanden ist [10]. Die Methode muss im Übrigen nach den Regeln der ärztlichen Kunst angewendet werden. Das bedeutet, dass die individuelle Anwendung am Patienten angesichts z. B. der Schwere der Erkrankung kunstgerecht erfolgen muss [11]. Es bedeutet hingegen nicht, dass der allgemeine Qualitätsanspruch, der in § 2 Abs. 1 S. 3 SGB V vor die Erstattungsfähigkeit von Leistungen gestellt wird, einzuhalten wäre. In diesem Kontext wird eine vorübergehende Absenkung der üblichen Qualitätsanforderungen zugunsten einer frühzeitigen Bereitstellung einer vielversprechenden, aber noch nicht vollständig evidenzbasierten Therapie in Kauf genommen. Diese Voraussetzungen können in Einzelfällen durchaus einen Kostenerstattungsanspruch für die Anwendung der Phagentherapie im Krankenhaus begründen.

Die Abrechnungsmöglichkeit der Phagentherapie im Krankenhaus erfordert eine Betrachtung der Zusatzentgelte und der NUB. Diese Instrumente ermöglichen es Krankenhäusern, spezifische Kosten außerhalb der DRG-Pauschalen zu decken. Zwar waren NUB-Anfragen beim Institut für das Entgeltsystem im Krankenhaus (InEK) für Reserveantibiotika bislang erfolglos, jedoch könnte die nun erfolgte Vergabe eines Operationen- und Prozedurenschlüssels (OPS) bei der Abbildung der tatsächlichen Kosten für zukünftige InEK-Kalkulationen hilfreich sein [12]. Nach ähnlichem Prinzip könnte dies auch für individuell hergestellte Phagenpräparate im Rahmen der magistralen Arzneimittelherstellung erfolgen.

#### Abrechnung über DRG-Pauschalen (§ 17b KHG).

Im DRG-System werden alle in Zusammenhang mit einer Diagnose anfallenden Leistungen und Therapien zu einer einheitlichen Fallpauschale gebündelt. Die damit vorgenommene Kostenerstattung umfasst gem. § 39 Abs. 1 S. 3 SGB V und § 2 Abs. 1 KHEntgG auch die Versorgung mit Arzneimitteln. Diese werden nicht gesondert im DRG-Katalog aufgeführt. Phagentherapeutika sind dementsprechend derzeit nicht spezifisch in den DRG-Katalogen berücksichtigt. Ihre Kosten können nur dann über die Fallpauschale abgedeckt werden, wenn sie als Bestandteil der definierten Standardleistungen anerkannt sind.

#### Neue Untersuchungs- und Behandlungsmethoden (NUB; § 6 Absatz 2 KHEntgG).

Für Leistungen oder Medikamente, die nicht im DRG-Katalog enthalten sind, können Krankenhäuser beim InEK einen Antrag auf ein krankenhausindividuelles Zusatzentgelt stellen. Dies erfolgt im Rahmen des sogenannten NUB-Verfahrens. Der Antrag muss jährlich gestellt und vom InEK positiv bewertet werden (Status 1). Eine positive Bewertung ermöglicht es den Krankenhäusern, die Kostenübernahme der Phagentherapie individuell mit den Krankenkassen zu verhandeln. Die Frage der zur Kostenerstattung erforderlichen medizinischen Notwendigkeit der neuen Methode richtet sich nach den Kriterien des § 2 Abs. 1a SGB V [13].

#### Zusatzentgelte (§ 17b Abs. 1 S. 7 KHG, § 9 Abs. 1 Nr. 2 KHEntgG).

Für Leistungen, die über den Umfang einer DRG hinausgehen, können Zusatzentgelte vereinbart werden, die im Entgeltkatalog separat ausgewiesen werden. Voraussetzung ist, dass die Leistung als medizinisch notwendig anerkannt wird und eine Vereinbarung über die Höhe des Zusatzentgelts zwischen Krankenhaus und Kostenträger erfolgt. Dies umfasst insbesondere auch die Kosten für bestimmte Arzneimittel. Hierunter wäre grundsätzlich die Phagentherapie zu subsumieren. Allerdings bleiben die Schwierigkeiten im Hinblick auf die fehlende Zulassung der Phagentherapeutika hier relevant.

Im ambulanten Sektor ist die Phagentherapie als Rezepturarzneimittel als neue Arzneimitteltherapie nur dann erstattungsfähig, wenn der G‑BA eine positive Nutzenbewertung nach § 135 SGB V vornimmt und eine entsprechende Richtlinie nach § 92 Abs. 1 S. 2 SGB V beschließt. Während einer Erprobung gem. § 137e Abs. 1 S. 1 SGB V kann die Leistung zulasten der GKV erbracht werden, sofern der G‑BA ihr das Potenzial einer erforderlichen Behandlungsalternative zuspricht. Eine regelhafte Erstattung setzt somit einen strukturierten Nachweis des diagnostischen und therapeutischen Nutzens, der medizinischen Notwendigkeit und der Wirtschaftlichkeit der Methode voraus [14].

### Notstandsversorgung § 2 Abs. 1a SGB V

Auf der Basis der Entscheidung des Bundesverfassungsgerichts vom 06.12.2005 [15] können unter umschriebenen Bedingungen Leistungen – auch Arzneimittelgaben – zulasten der GKV erbracht werden, die nicht allgemein anerkannt bzw. zur Kostenerstattung zugelassen sind. Hier kommt es entgegen der Potenzialerstattung nach § 137c SGB V nicht darauf an, ob die Methode generell das Potenzial einer erforderlichen Behandlungsalternative hat.

Phagentherapien sind bisher nicht als reguläre GKV-Leistungen anerkannt. Unter bestimmten Voraussetzungen, wie im Rahmen der sog. Notstandsverordnung, können jedoch die Kosten für nicht zugelassene Arzneimittel dennoch zulasten der GKV abgerechnet werden. Dies gilt, wenn kein geeignetes zugelassenes Arzneimittel verfügbar ist, alle zugelassenen Therapien ausgeschöpft sind und die Erkrankung für den Patienten lebensbedrohlich ist [16].

Nach ständiger Rechtsprechung des BSG und des BVerfG ist eine Erkrankung als lebensbedrohlich oder regelmäßig tödlich einzustufen, wenn sie in überschaubarer Zeit das Leben beenden kann und dies eine notstandsähnliche Situation herbeiführt, in der Versicherte nach allen verfügbaren medizinischen Hilfen greifen müssen. Nach den konkreten Umständen des Falles muss bereits drohen, dass sich mit hoher Wahrscheinlichkeit der voraussichtlich tödliche Krankheitsverlauf innerhalb eines kürzeren, überschaubaren Zeitraums verwirklichen wird, sodass Versicherte nach allen verfügbaren medizinischen Hilfen greifen müssen (st. Rspr.; vgl. BVerfG vom 11.04.2017 – 1 BvR 452/17 [17] – SozR 4‑2500 § 137c Nr. 8 Rdnr. 25 [18]; vgl. BSG vom 29.06.2023 – B 1 KR 35/21 R [19] – Rdnr. 21 und 22 m. w. N. BSG, a. a. O., Rn. 27).

Für Phagentherapeutika könnte ein solcher Ausnahmefall beispielsweise bei einem Infekt mit multiresistenten Keimen vorliegen, wenn keine wirksamen Reserveantibiotika mehr vorliegen. Dabei müsste die Infektion potenziell lebensbedrohlich sein. Nach der Rechtsprechung ist das Kriterium der Lebensbedrohlichkeit erfüllt, wenn die Krankheit jederzeit in einen tödlichen, das heißt binnen kürzerer Zeit zum Tode führenden Verlauf umschlagen kann. Das BSG hat zuletzt geurteilt, dass dies grundsätzlich auch bei einer Erkrankung angenommen werden kann, die möglicherweise noch eine Lebenserwartung von 2 Jahren bedeutet [8]. Ein solches Szenario ist bei Infektionen mit resistenten Bakterien durchaus gegeben. Dies verdeutlicht ein Urteil des Sozialgerichts Detmold, in dem die Kostenerstattung für eine Behandlung mit individuell hergestellten Bakteriophagen bei einem schweren Harnwegsinfekt anerkannt wurde. Das Gericht führte aus, dass der Klägerin nicht zugemutet werden könne, die erheblichen Gesundheitsrisiken einer Nierenbeckenentzündung und Urosepsis in Kauf zu nehmen [20].

## Sollanalyse zur regelhaften Kostenerstattung in Deutschland

Phagentherapeutika werden derzeit weder im stationären noch im ambulanten Sektor regelhaft vergütet und zählen nicht zum medizinischen Standard aufgrund noch fehlender Evidenz. Ihre Einführung ist mit wirtschaftlichen Herausforderungen wie Vorfinanzierung, Refinanzierungsunsicherheiten und regulatorischen Hürden verbunden. Eine nachhaltige Integration erfordert daher eine differenzierte Betrachtung der Vergütungsmodelle je nach Herstellungsverfahren. Die Vergütungshöhe müsste in einem weiteren Schritt diskutiert werden, was nicht Gegenstand des vorliegenden Beitrags ist; zumindest sollte sich diese an dem zu erwartenden Zusatznutzen orientieren.

### Phagen als Rezepturarzneimittel

Die Vergütungsmechanismen der CAR-T-Zell-Therapie und der Stammzelltherapie bieten Anhaltspunkte für die Integration der Phagentherapie als individualisierte Rezepturarzneimittel in die Krankenhausvergütung. Insbesondere bei der CAR-T-Zell-Therapie zeigen sich Herausforderungen, die auch für die Phagentherapie relevant sein könnten: Hohe Kosten und der erhebliche Koordinationsaufwand zwischen Zentrum und Hersteller führen zu Mehraufwendungen, die bislang nicht vollständig im Vergütungssystem abgebildet werden (vgl. [21]). Analog zur CAR-T-Zell-Therapie könnte die stationäre Phagentherapie über das DRG-System erfolgen, basierend auf Fallpauschalen für schwere Infektionen und Sepsis. Aufgrund der hohen Wirtsspezifität der Phagentherapie wäre die Etablierung eines NUB-Entgelts für die patientenspezifische Phagenherstellung sinnvoll, um Kosten für mikrobiologische Analysen, Selektion und Produktion abzudecken. Ergänzend könnte eine arzneimittelspezifische Zusatzvergütung erfolgen, ähnlich wie bei der CAR-T-Zell-Therapie, wo nicht nur das Arzneimittel selbst, sondern auch der zusätzliche stationäre Mehraufwand vergütet wird. In lebensbedrohlichen Fällen wären zusätzliche Vergütungen für intensivmedizinische Maßnahmen erforderlich. Auch Transport- und Logistikkosten bei externer Herstellung sollten in die Vergütungsstruktur integriert werden. Die Kombination aus DRG-Pauschalen, NUB-Entgelten und Zusatzvergütungen würde eine wirtschaftliche Einbettung der Phagentherapie in die Krankenhausvergütung ermöglichen und ihre klinische Anwendung fördern.

Aus ökonomischer Perspektive wäre eine standardisierte Herstellung von Phagentherapeutika mit nachgewiesener Wirksamkeit und Unbedenklichkeit als sog. Wildtypphagen in dafür zugelassenen Herstellbetrieben und Phagenbanken zu bedenken. Ergänzend könnten standardisierte, bewährte magistrale Rezepturen oder sog. Monografien im Arzneibuch festgehalten werden. Diese würden Apotheken und Krankenhausapotheken die Herstellung spezifischer Phagenmischungen ermöglichen.

Ähnlich wie bei der CAR-T-Zell-Therapie könnte die Phagentherapie als Rezepturarzneimittel zunächst auf spezialisierte Zentren beschränkt werden. Trotz unterschiedlicher Anforderungen an Nebenwirkungsmanagement und Behandlungsprozess könnte eine Zentralisierung mit Bündelung der Fallzahlen aufgrund der benötigten Infrastruktur, Qualifikation und Organisation sinnvoll sein.

### Phagen als Fertigarzneimittel

Sollten Phagen als industriell hergestellte Fertigarzneimittel zulasten der GKV verordnet werden können, muss der pharmazeutische Unternehmer hierfür zunächst die Zulassung erhalten und dann die frühe Nutzenbewertung entsprechend § 35a SGB V gemäß den Bestimmungen des Arzneimittelmarktneuordnungsgesetzes (AMNOG) durchlaufen. Für die Zulassung müssen pharmazeutische Qualität, Sicherheit und Wirksamkeit im Rahmen von klinischen Studien nachgewiesen sein. Derzeit sind in Deutschland jedoch keine Phagenarzneimittel zugelassen. Da Phagen gezielt gegen spezifische Bakterienstämme wirken, bestehen oft nur kleine Patientenpopulationen, was die Durchführung randomisierter, kontrollierter Studien erschwert. Derartige Studien wären auch für den Nachweis des Zusatznutzens gegenüber der bestehenden Standardtherapie erforderlich, die momentan fehlen. Um dennoch Innovationen den Weg zu bereiten, könnten Instrumente wie ein dynamischer Evidenzpreis eingeführt werden. Hierfür könnte sich die Vergütungshöhe zunächst an den Preisen der Regelversorgung orientieren; sobald über eine anwendungsbegleitende Datenerhebung mehr Evidenz für die neuartige Therapie vorliegt, sollte der Preis anhand des Zusatznutzens angepasst werden. Alternativ hierzu könnten erfolgsabhängige Vergütungsmodelle („outcome-based payments“) etabliert werden, um im Sinne eines sog. Risk-Sharing zwischen pharmazeutischem Unternehmer bzw. Anbieter der Phagentherapie und dem Kostenträger die Vergütung im Zeitverlauf an den Behandlungserfolg anzuknüpfen.

Die Vergütung von Phagentherapeutika im stationären Bereich könnte bis dahin wie erläutert über Zusatzentgelte (§ 6 Abs. 1 KHEntgG) oder im Rahmen des NUB-Verfahrens (§ 6 Abs. 2 KHEntgG) erfolgen.

## Ausblick und Empfehlung

Die erfolgreiche Integration neuer Therapien wie der Phagentherapie in den klinischen Alltag erfordert eine umfassende strategische Vorbereitung und Prozessoptimierung. Dabei sind folgende Schritte bedeutsam:

### Evidenzbasierte Vorbereitung.

Der Nachweis von Wirksamkeit und Sicherheit durch klinische Studien für spezifische Indikationen bildet die unabdingbare Grundlage für die Integration der Phagentherapie. Diese Studien sind unerlässlich, um eine spätere Zulassung und Kostenerstattung zu ermöglichen. Auch die alternativen Erstattungsmöglichkeiten erfordern jedenfalls Wirksamkeitsnachweise. Die Zulassung als neue Behandlungsmethode erfordert eine positive G‑BA-Richtlinie, die zumindest die Erprobung gem. § 137 e SGB V gestattet. Gesundheitsökonomische Analysen, die langfristige Einsparungen durch die Vermeidung schwerwiegender Infektionen analysieren, sind wichtiger Bestandteil der strategischen Planung. Sie helfen, den Nutzen der Therapie nachvollziehbar darzustellen.

### Optimierung von Vergütungsanträgen.

Die rechtzeitige Beantragung von Zusatzentgelten und NUB erfordert die Einhaltung der jährlichen Fristen des InEK (bis Ende Oktober). Die Nutzung vorhandener Datenbanken zur Dokumentation von Kosten und Nutzen kann den Antragsprozess unterstützen und beschleunigen.

### Strategische Abstimmung mit Kostenträgern.

Die erfolgreiche Einführung der Phagentherapie erfordert eine Zusammenarbeit mit den Kostenträgern, insbesondere bei der Verhandlung von Zusatzentgelten und NUB auf einer evidenzbasierten Grundlage.

Derzeit stellen die begrenzte Evidenz zur Wirksamkeit der Phagentherapie sowie ihre Vergütung nur in Ausnahmefällen ein Hemmnis für die wirtschaftliche Attraktivität dar – sowohl für pharmazeutische Unternehmen als auch für Krankenhäuser. Um diese Barrieren zu überwinden, sind gezielte staatliche Forschungsförderung, die Unterstützung von Start-ups sowie Vergütungsmodelle erforderlich, die den Besonderheiten innovativer Therapien mit kleinen Patientengruppen und zunächst begrenzter Evidenz Rechnung tragen.

## Fazit

Die Vergütung der Phagentherapie steht vor einer doppelten Herausforderung: Industriell hergestellte Phagentherapeutika könnten eine Zulassung und potenzielle Erstattung nach SGB V erhalten, doch die hohe Wirtsspezifität erschwert eine ausreichende Patientenzahl. Diese ist erforderlich, um für Zulassung und Nutzenbewertung hinreichend Evidenz darzustellen, aber auch wirtschaftliche Anreize für pharmazeutische Unternehmen zu schaffen. Daher könnten öffentliche Fördermaßnahmen wie Forschungsprogramme, garantierte Abnahmemengen oder steuerliche Anreize nötig sein. Gleichzeitig bleibt die Erstattung individuell hergestellter Phagentherapeutika ungelöst, da Vergütungsmöglichkeiten im stationären Sektor geschaffen werden müssten, die sich nicht nur auf die Versorgung von schwerkranken Patienten fokussieren. Bestehende Ausnahmeregelungen im stationären und ambulanten Sektor sind durch Begründungsaufwand und Einzelfallentscheidungen geprägt. Ein evidenzbasiertes Erstattungsmodell mit dynamischer Nutzenbewertung und alternativen Finanzierungswegen wie Risk-Sharing könnte den Zugang zur Phagentherapie verbessern, ohne wissenschaftliche Evidenz zu vernachlässigen.
